# Nationwide study in France investigating the impact of diabetes on mortality in patients undergoing abdominal aortic aneurysm repair

**DOI:** 10.1038/s41598-021-98893-x

**Published:** 2021-09-29

**Authors:** Juliette Raffort, Fabien Lareyre, Roxane Fabre, Ziad Mallat, Christian Pradier, Laurent Bailly

**Affiliations:** 1 Université Côte d’Azur, CHU, Inserm U1065, C3M, Nice, France; 2 Institute 3IA Côte d’Azur, Université Côte d’Azur, Nice, France; 3 Department of Vascular Surgery, Hospital of Antibes Juan-Les-Pins, Antibes, France; 4 CoBTeK Lab, Université Côte d’Azur, Nice, France; 5 Division of Cardiovascular Medicine, University of Cambridge, Cambridge, UK; 6 Public Health Department, University Hospital of Nice, Université Côte d’Azur, Nice, France

**Keywords:** Cardiovascular diseases, Vascular diseases, Aneurysm

## Abstract

The aim of this nationwide study was to analyze the impact of diabetes on post-operative mortality in patients undergoing AAA repair in France. This 10-year retrospective, multicenter study based on the French National electronic health data included patients undergoing AAA repair between 2010 and 2019. In-hospital post-operative mortality was analyzed using Kaplan–Meier curve survival and Log-Rank tests. A multivariate regression analysis was performed to calculate Hazard Ratios. Over 79,935 patients who underwent AAA repair, 61,146 patients (76.5%) had at least one hospital-readmission after the AAA repair, for a mean follow-up of 3.5 ± 2.5 years. Total in-hospital mortality over the 10-year study was 16,986 (21.3%) and 4581 deaths (5.8%) occurred during the first hospital stay for AAA repair. Age over 64 years old, the presence of AAA rupture and hospital readmission at 30-day were predictors of post-operative mortality (AdjHR = 1.59 CI 95% 1.51–1.67; AdjHR = 1.49 CI 95% 1.36–1.62 and AdjHR = 1.92, CI 95% 1.84–2.00). The prevalence of diabetes was significantly lower in ruptured AAA compared to unruptured AAA (14.8% vs 20.9%, P < 0.001 for type 2 diabetes and 2.5% vs 4.0%, P < 0.001 for type 1 diabetes). Type 1 diabetes was significantly associated with post-operative mortality (AdjHR = 1.30 CI 95% 1.20–1.40). For type 2 diabetes, the association was not statistically significant (Adj HR = 0.96, CI 95% 0.92–1.01). Older age, AAA rupture and hospital readmission were associated with deaths that occurred after discharge from the first AAA repair. Type 1 diabetes was identified as a risk factor of post-operative mortality. This study highlights the complex association between diabetes and AAA and should encourage institutions to report long-term follow-up after AAA repair to better understand its impact.

## Introduction

Cardiovascular diseases are the leading cause of premature death in developed countries^1^. Among them, abdominal aortic aneurysm (AAA), defined as a focal dilatation of the aorta, has become a significant public health challenge worldwide, with extremely high rates of mortality in case of rupture^2–4^. Its prevalence in European countries has been estimated between 1.3 to 3.3% of men over 65 years and 0.7% of women over 60 years^2,5^. The treatment of AAA relies on surgical approaches and can be performed using open repair or endovascular aneurysm repair (EVAR)^2,4,6^.

AAA is often associated with atherosclerosis and cardiovascular risk factors such as age, male sex, tobacco use, hypertension or obesity {Wanhainen, 2019 #4}{Kent, 2010 #67}. Intriguingly, epidemiological studies have pointed to a negative association between diabetes and AAA^7^. Diabetic patients develop smaller aneurysm compared to non-diabetic subjects^8,9^. The prevalence, the incidence, the aneurysmal growth as well as the risk of rupture is lower in diabetic patients^8,10–12^. While diabetes may play a protective role on aneurysm development^7^, its effect on the post-operative outcomes in patients undergoing AAA repair is more controversial and is not well-established^13–18^.

A better understanding of the impact of diabetes on the outcomes of patients undergoing AAA repair would help to improve their management and anticipate post-operative complications.

In this 10-year descriptive nationwide study, we aimed to explore the association between diabetes and post-operative mortality in patients undergoing AAA repair in France.

## Methods

### Study design and data collection

This population-based, multicenter, cohort study was based on the French National Health insurance information system and was conducted in accordance with the French National Health Data Institute regulation (Institut National des Données de Santé, INDS). The protocol was approved by the Institutional Review Board of the University Hospital of Nice and by the French National Health Information System (Système National des Données de Santé, SNDS). This electronic database has been previously described^19,20^ and informed consent was waived for this type of study in accordance with the French National Health Data Institute regulation. Information was extracted from anonymous discharge reports completed and coded at the end of each hospital stay.

Eligible patients where those undergoing an AAA repair for the first time in any public or private hospital in France between 2010 and 2019. AAA repair was classified into open repair or EVAR according to the Common Classification of Medical Acts (CCAM). Collected data included the general characteristics of patients, the comorbidities, the type of AAA repair, the presence of an aneurysm rupture or not, and the post-operative outcomes (mortality and in-hospital readmission at 30 days). Comorbidities were defined according to the International Classification of Diseases (ICD-10). The codes used are presented in the Supplementary data.

The all-cause mortality was analyzed based on the French National electronic health data that allowed to identify all deaths registered during a hospital stay over a ten-year period. The in-hospital mortality during the first AAA repair was analyzed. Hospital readmission at 30 days included all re-hospitalization occurring within 30 days after discharge from the first AAA repair whatever the cause. For the follow-up, patients who had at least one re-admission at hospital after the first AAA repair recorded in the National French electronic health data were included. In-hospital total mortality was defined as the number of deaths during hospital stay recorded from the first AAA repair until the last follow-up. The French National Health Information System only had access and recorded deaths occurring during a hospital stay. The analysis thus excluded deaths occurring out of hospital.

### Statistical analysis

Results for quantitative variables were expressed as means ± standard deviation (SD) and as percentages for categorical variables. Group differences were analyzed using Student t-test for quantitative variables and Chi-2 test or Fisher test for qualitative variables.

A survival analysis was performed in order to identify risk factors associated with post-operative mortality. Only patients who had at least one follow-up at hospital after the first AAA repair recorded in the National French electronic health data between 2010 and 2019 were included. The release date from the hospital after the first AAA repair was considered as the starting point and the follow-up was calculated from this date until the last hospital stay. The analysis excluded deaths that occurred during the hospital stay for the first AAA repair. Kaplan–Meier curve survival and Log-Rank tests were used to analyze the risk of post-operative mortality. Multivariate regression analysis using a Cox proportional model was performed to calculate Hazard Ratios with 95% CI. The hypothesis of the proportional risk was evaluated according to the survival curves using the double negative logarithm function. The factors which did not comply with the hypothesis of the proportional risk were excluded from the model. A two-tailed P-value < 0.05 was considered as statistically significant. Statistical analysis was performed using SAS Enterprise 5.1 (SAS Institute, Cary, NC).

### Ethical approval for research

The study was conducted in accordance with the French National Health Data Institute regulation.

## Results

### Characteristics of patients who underwent AAA repair in France between 2010 and 2019

**Figure 1:**
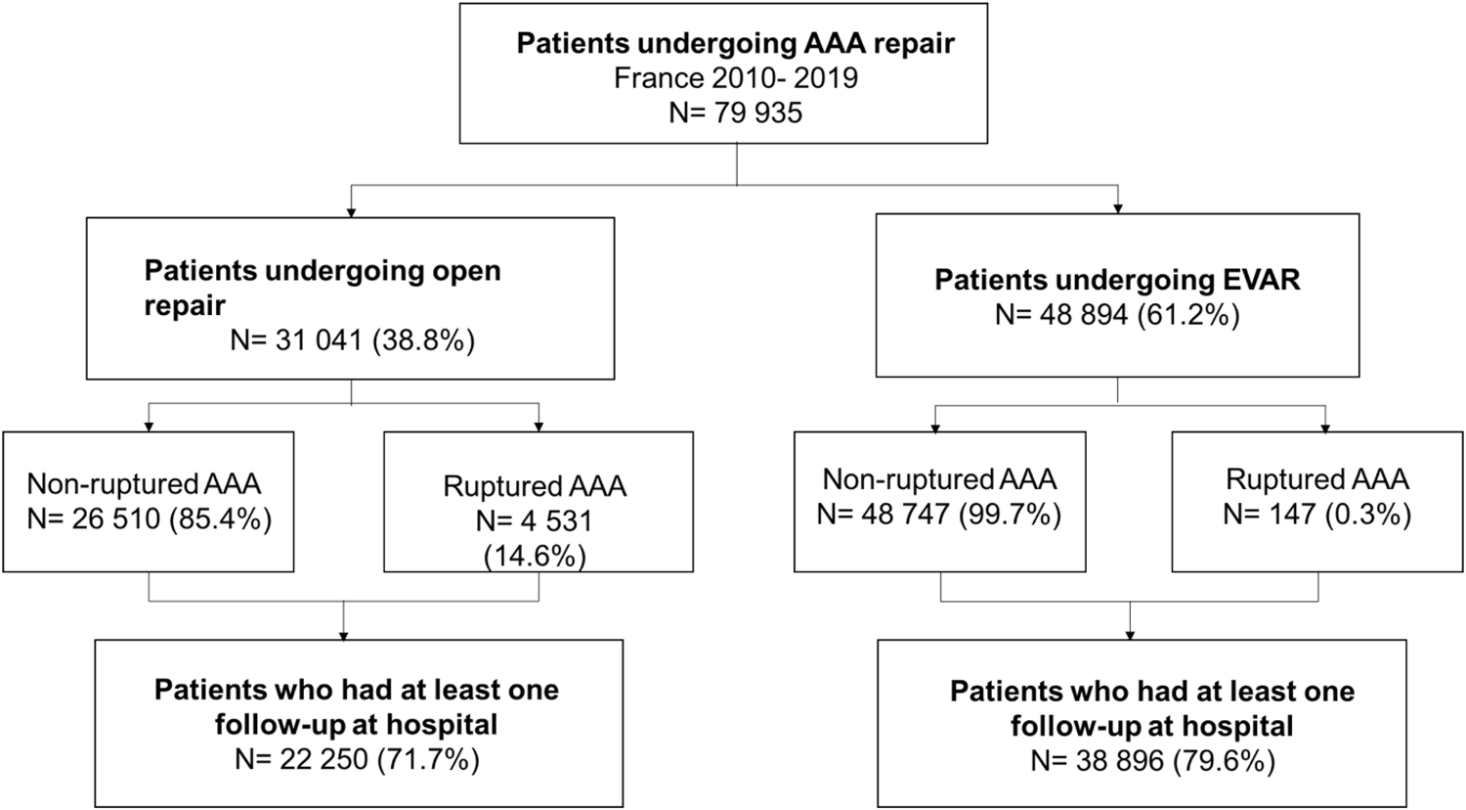
Flow chart of the study population. Data are expressed as n (%).

Between 2010 and 2019, 79,935 patients underwent AAA repair in France and were included in this study; 31,041 (38.8%) had an open repair while 48,894 (61.2%) underwent EVAR. The flow chart of the study population is depicted in Fig. 1. The mean age of the population was 71.6 ± 9.7 years and 90.6% were men (Table 1). The in-hospital death during the first AAA repair was 4581 (5.8%). The total of in-hospital death over the 10-year study was 16,986 (21.3%). It included deaths registered at the hospital from the first AAA repair until the last follow-up.

**Table 1:** Characteristics of patients operated for an abdominal aortic aneurysm classified according to the type of surgery and the presence of a rupture.

	Total N = 79,935	Open repair N = 31,041	EVAR N = 48,894	p	Non ruptured AAA N = 75,257	Ruptured AAA N = 4678	p
Age^y^	71.6 ± 9.7	68.7 ± 9.3	73.4 ± 9.1	< 0.001	71.6 ± 9.7	72.1 ± 10.4	< 0.001
Sex ratio M/F (%)	90.6/9.4	90.8/9.2	90.4/9.6	0.09	90.7/9.3	88.8/11.2	< 0.001
In-hospital stay^d^	10.0 ± 11.1	13.9 ± 13.9	7.5 ± 7.9	< 0.001	9.7 ± 10.2	15.5 ± 20.0	< 0.001
Rupture	4678 (5.9)	4531 (14.6)	147 (0.3)	< 0.001			
In-hospital death during the first AAA repair	4581 (5.8)	3324 (10.7)	1257 (2.6)	< 0.001	2708 (3.6)	1873 (40.0)	< 0.001
In-hospital total death	16,986 (21.3)	7143 (23.0)	9843 (20.1)	< 0.001	14,561 (19.4)	2425 (51.8)	< 0.001
Arterial hypertension	60,897 (76.2)	23,283 (75.0)	37,614 (76.9)	< 0.001	57,853 (76.9)	3044 (65.1)	< 0.001
Dyslipidemia	40,989 (51.3)	15,895 (51.2)	25,094 (51.3)	0.75	39,504 (52.5)	1485 (31.7)	< 0.001
T2D	16,408 (20.5)	5931 (19.1)	10,477 (21.4)	< 0.001	15,718 (20.9)	690 (14.8)	< 0.001
T1D	3092 (3.9)	1049 (3.4)	2043 (4.2)	< 0.001	2973 (4.0)	119 (2.5)	< 0.001
Smoking	19,225 (24.1)	8964 (28.9)	10,261 (21.0)	< 0.001	18,238 (24.2)	987 (21.1)	< 0.001
Obesity	17,541 (21.9)	6352 (20.5)	11,189 (22.9)	< 0.001	16,738 (22.2)	803 (17.2)	< 0.001
Obstructive sleep apnea	8403 (10.5)	2769 (8.9)	5634 (11.5)	< 0.001	8105 (10.8)	298 (6.4)	< 0.001
CHF	28,499 (35.7)	10,170 (32.8)	18,329 (37.5)	< 0.001	27,138 (36.1)	1361 (29.1)	< 0.001
Chronic respiratory disease	23,096 (28.9)	9075 (29.2)	14,021 (28.7)	0.09	22,051 (29.3)	1045 (22.3)	< 0.001
Chronic kidney disease	16,332 (20.4)	5561 (17.2)	10,771 (22.0)	< 0.001	15,487 (20.6)	845 (18.1)	< 0.001
Stroke	14,959 (18.7)	2394 (7.7)	4403 (9.0)	< 0.001	14,337 (19.1)	622 (13.3)	< 0.001

Results are expressed as mean ± SD or n (%).

*AAA* abdominal aortic aneurysm, *CHF* congestive heart failure, *d* days, *EVAR* endovascular aneurysm repair, *F* female, *M* male, *T1D* type 1 diabetes, *T2D* type 2 diabetes, *y* years.

In 94.1% of cases, the AAA was not ruptured (n = 75,257), while ruptured aneurysms concerned less than 6% of patients (n = 4678; 5.9%). Most of the patients with ruptured AAA were treated using open repair (4531 out of 4678, 96.8%). The total in-hospital mortality was significantly higher in patients with AAA rupture (2425, 51.8%) compared to those without rupture (14,561, 19.4%, P < 0.001). The in-hospital mortality during the first hospital stay for AAA repair was significantly higher in patients who had open repair compared to those who underwent EVAR (10.7% vs 2.6%, P < 0.001). In case of AAA rupture, the death occurred more frequently during the hospital stay of the first AAA repair (1873 out of 2425, 77.2%) while it represented only 18.7% (2708 out of 14,561) of the total deaths in patients without rupture.

The prevalence of type 2 diabetes was 16,408 patients (20.5%) and 3092 patients (3.9%) for type 1 diabetes. The prevalence of followed comorbidities was significantly lower in patients with AAA rupture compared to those without rupture. This included type 2 diabetes (14.8% vs 20.9%, P < 0.001), type 1 diabetes (2.5% vs 4.0%, P < 0.001), arterial hypertension (65.1%, vs 76.9%, P < 0.001), dyslipidemia (31.7% vs 52.5%, P < 0.001), obesity (17.2% vs 22.2%, P < 0.001) or obstructive sleep apnea (6.4% vs 10.8%, P < 0.001). The prevalence of known cardiovascular risk factors and followed diseases was also lower, with a lower rate of smoking (21.1% vs 24.2%, P < 0.001), congestive heart failure (29.1% vs 36.1%, P < 0.001) or past history of stroke (13.3% vs 19.1%, P < 0.001).

### Association between diabetes and post-operative mortality of patients who underwent AAA repair

Post-operative mortality after AAA repair was compared between diabetic and non-diabetic patients and was analyzed according to the surgical technique and the presence or absence of rupture (Table 2). In patients undergoing AAA open repair, the in-hospital mortality during the first AAA repair was significantly lower in diabetic patients compared to non-diabetic (4.5% vs 5.8%, p < 0.01 for unruptured AAA and 32.1% vs 42.6%, p < 0.01 for ruptured AAA). However, the total in-hospital mortality was significantly higher in diabetic patients compared to non-diabetic after open repair for unruptured AAA (20.5% vs 17.5, p < 0.01). In case of ruptured AAA treated with open repair, diabetic patients had lower total in-hospital mortality (47.4% vs 52.5%, p < 0.05). For patients undergoing EVAR, no significant difference was observed for in-hospital death during the first AAA repair. The in-hospital total mortality was higher in diabetic patients after treatment of unruptured AAA (21.9% vs 19.5%, p < 0.01) while there was no significant difference for ruptured AAA.

**Table 2:** Post-operative mortality of patients with and without diabetes who underwent abdominal aortic aneurysm repair according to surgical technic and presence or absence of ruptured AAA.

	Open repair						EVAR*					
	No AAA rupture			AAA rupture			No AAA rupture			AAA rupture		
	Diabetes N = 5271	No diabetes N = 21,239	p	Diabetes N = 660	No diabetes N = 3871	p	Diabetes N = 10,447	No diabetes N = 38,300	p	Diabetes N = 30	No diabetes N = 117	p
In-hospital death during the first AAA repair % (N)	4.5 (239)	5.8 (1224)	< 0.01	32.1 (212)	42.6 (1649)	< 0.01	6.6 (218)	6.9 (1027)	0.36	6.7 (2)	8.6 (10)	1.0**
In-hospital total death % (N)	20.5 (1079)	17.5 (3720)	< 0.01	47.4 (313)	52.5 (2031)	< 0.05	21.9 (2289)	19.5 (7473)	< 0.01	56.7 (17)	54.7 (64)	0.85

* Endovascular aneurysm repair ** Fisher exact Test.

*AAA* abdominal aortic aneurysm.

### Factors associated with post-operative mortality after AAA repair

In total, 61,146 patients (76.5%) had at least one follow-up after the release date from the first AAA repair and were included in the analysis (Fig. 1). The mean follow-up was 3.5 years± 2.5. A multivariate analysis was performed to identify risk factors of post-operative mortality. The analysis excluded in-hospital mortality during the first AAA repair. The total of in-hospital deaths that occurred after the first AAA repair was 12,400 (20.3%) including 3817 deaths after open repair and 8583 deaths after EVAR. Age over 64 years old and the presence of AAA rupture were positively associated with the risk of in-hospital post-operative total mortality (AdjHR = 1.59 CI95% = 1.51–1.67 and AdjHR = 1.49 CI95% = 1.36–1.62) (Table 3). In-hospital readmission at 30-day was a strong predictor of in-hospital post-operative mortality (AdjHR = 1.92, CI95% = 1.84–2.00). Compared to open repair, EVAR was associated with increased risk of in-hospital post-operative mortality (AdjHR = 1.48, CI95% 1.42–1.54). Among cardiovascular diseases, congestive heart failure and history of stroke were significantly associated with the risk of in-hospital post-operative mortality (Adj HR. = 1.40, CI95% = 1.35–1.46 and AdjHR = 1.19, CI95% = 1.14–1.23, respectively). Other comorbidities such as chronic respiratory disease or chronic kidney disease were also predictive of in-hospital post-operative mortality. Type 1 diabetes was positively associated with the risk of post-operative mortality (AdjHR = 1.30, CI95% = 1.20–1.40). For type 2 diabetes, the association was not statistically significant (Adj HR = 0.96, CI95% = 0.92–1.01).

**Table 3:** Risk factors of in-hospital mortality after abdominal aortic aneurysm surgical repair.

	Total* N = 61,146		Open repair* N = 22,250		EVAR* N = 38,896	
	Adjusted hazard ratio	CI 95%	Adjusted hazard ratio	CI 95%	Adjusted hazard ratio	CI 95%
In-hospital readmission at 30 days	1.92	1.84–2.00	2.03	1.88–2.19	1.88	1.79–1.98
Age > 64 years	1.59	1.51–1.67	1.53	1.42–1.65	1.63	1.52–1.74
Ruptured AAA	1.49	1.36–1.62	1.48	1.34–1.63	1.60	1.26–2.02
EVAR (ref. open repair)	1.48	1.42–1.54				
CHF	1.40	1.35–1.46	1.33	1.24–1.42	1.44	1.38–1.51
Chronic respiratory disease	1.33	1.28–1.38	1.35	1.26–1.45	1.32	1.26–1.38
Chronic kidney disease	1.31	1.26–1.36	1.30	1.21–1.39	1.31	1.25 -1.37
T1D	1.30	1.20–1.40	1.33	1.16–1.52	1.28	1.17–1.41
Stroke	1.19	1.14–1.23	1.21	1.12–1.30	1.18	1.12–1.23
Female sex	1.06	1.00–1.13	1.15	1.03–1.28		
Smoking	1.06	1.02–1.11	1.13	1.06–1.22		
Dyslipidemia	0.72	0.69–0.75	0.75	0.71–0.81	0.71	0.68–0.74
Obesity	0.83	0.79–0.86	0.79	0.71–0.89	0.84	0.80–0.89
Obstructive sleep apnea	0.83	0.79–0.86	0.81	0.74–0.88	0.85	0.79–0.91
Arterial hypertension	0.96	0.92–1.01	0.93	0.85–1.02	0.97	0.92–1.03
T2D	0.96	0.92–1.01	1.00	0.93–1.09	0.95	0.90–1.00

*Population of patients who had at least one follow-up i.e. admission at the hospital after the AAA surgical repair.

Results are expressed as mean ± SD or n (%).

*AAA* abdominal aortic aneurysm, *CHF* congestive heart failure, *EVAR* endovascular aneurysm repair, *T2D* type 2 diabetes, *T1D* type 1 diabetes.

## Discussion

This ten-year observational nationwide cohort of 79,935 patients reports for the first time the outcomes of patients from the French National Health data and represents one of the largest European retrospective study on AAA repair^21–23^.

The proportion of patients who had EVAR/ open repair was balanced (approximatively 0.6) and was similar to the practice observed in other European countries^21,22^. Ruptured AAA were most often treated using open repair (96.2% of cases). A population-based study in Finland between 2000 and 2014 revealed similar results, with 1627 treated with open repair over the 1687 ruptured AAA (96.4%). Several observational and multicenter randomized clinical trials have demonstrated improved outcomes after EVAR for ruptured AAA^4,24^ and national trends in the United States confirmed that EVAR is increasingly used in the treatment of AAA rupture^25,26^. Changes in the management of ruptured AAA are thus to be expected in France within the next decade.

The in-hospital mortality of patients operated during the first hospital stay for a non-ruptured AAA in France was 3.6%. A nationwide analysis of 84,631 patients in Germany reported comparable rate, with 3.3% of in-hospital mortality in patients operated for intact AAA^21^. In case of aortic rupture, the in-hospital mortality during the first hospital stay for AAA repair was high (40%) and was concordant with other published studies^27,28^.

The prevalence of diabetes (including type 1 and type 2) was 24.4%, which corresponds to a high range compared to other cohorts previously published^17,18,29–31^. In our cohort, the prevalence of diabetes was significantly lower in patients with ruptured AAA compared to non-ruptured. This is concordant with a meta-analysis of 11 studies that showed that diabetes was associated with significantly lower prevalence/incidence of AAA rupture (OR/ HR = 0.77; CI95% = 0.63–0.95, P = 0.01)^12^. Nevertheless, we also found that the prevalence of the others cardiovascular and metabolic comorbidities (such as arterial hypertension, dyslipidemia, obesity, obstructive sleep apnea, smoking, congestive heart failure or past history of stroke) was significantly lower in patients with ruptured AAA compared to non-ruptured AAA. The paradoxical negative association observed in our study could be explained by a delay in the diagnosis of AAA in patients who did not have followed or known cardiovascular and metabolic comorbidities. It can be hypothesized that patients with comorbidities benefit from a closer follow-up and a screening of associated cardiovascular diseases. AAA in these patients may be discovered at earlier, asymptomatic and non-ruptured stage. On the other hand, it can be assumed that most of the patients who presented a rupture may have not been previously diagnosed for an AAA and may have not benefited from a systematic screening of cardiovascular comorbidities. It is thus possible that cardio-metabolic comorbidities may be underdiagnosed and underestimated in patients admitted for a ruptured AAA.

Interestingly, we found that in-hospital mortality during the first AAA repair was lower in diabetic patients compared to non-diabetic after AAA open repair for both ruptured and unruptured AAA. There was no significant difference for EVAR. However, in-hospital total mortality was higher in diabetic patients treated with open repair for an unruptured AAA. This result is corroborated by a systematic review including 64 studies that investigated the association between diabetes and AAA^16^. The authors found that diabetic patients showed lower survival rates at 2 to 5-year follow-up compared to non-diabetic patients^16^.

Diabetic patients undergoing EVAR for unruptured AAA had also higher in-hospital total mortality compared to non-diabetic. Similar results were observed from the EUROSTAR registry that included 6017 patients undergoing EVAR^17^ were mortality was significantly higher in the diabetic population compared to non-diabetic patients (13% vs 10%, p < 0.039; OR = 1.27, CI95% = 1.01–1.59)^17^.

Taken together, our results suggest that in non-ruptured AAA treated with open repair and EVAR, the presence of diabetes is not associated with worsen immediate post-operative outcomes but it may impair survival on longer follow-up period. The survival analysis and results on long-term mortality showed a worse prognosis in patients with type 1 diabetes after both open repair and EVAR, while type 2 diabetes was not identified as a significant risk factor. Other cardiovascular or metabolic comorbidities including the presence of congestive heart failure, past history of stroke, or smoking were identified as risk factors of post-operative mortality. Other studies have also identified heart failure, ischemic heart disease, cerebrovascular disease and diabetes as risk factors of mortality following elective AAA repair^32,33^. Nevertheless, we found in our cohort that the total mortality was significantly lower in patients presenting followed dyslipidemia, obesity, obstructive sleep apnea or smoking compared to those who did not have these comorbidities. Note that non-smokers were significantly older than smokers and could explain this result. Johal et al. recently aimed to examine patterns of 10-year survival after elective repair of unruptured AAA and investigated the survival among patients of different age and different co-morbidity score profiles from the English National Health register^34^. The long-term survival patterns after elective open repair and EVAR for unruptured AAA varied markedly across patients with different age and co-morbidity profiles. This underlines the complex impact of comorbidities on the post-operative outcomes and suggests that multiparametric predictive scores would be of interest.

The survival analysis after discharge from the first AAA repair revealed that aging, the presence of an aortic rupture, in-hospital readmission at 30 days, chronic respiratory and kidney diseases were predictive factors of post-operative mortality. These results are concordant with a meta-analysis including 45 studies investigating factors influencing survival following AAA repair^35^. End stage renal disease, chronic obstructive pulmonary disease, age, cardiac failure, cerebrovascular diseases were associated with poor long-term survival. In our cohort, we found that in-hospital mortality was higher in patients who had open repair, and could be partially explained by the higher proportion of ruptured AAA. However, EVAR was associated with increased risk of long-term mortality after discharge from the hospital. Randomized clinical trials have suggested that EVAR was associated with early survival gain, while open repair showed similar or late survival benefits^36–39^.

### Limitations

This is a retrospective observational study based on electronic administrative database and the results may depend on the coding system. Nevertheless, the French National insurance information system uses codes based on standardized definitions. Data are extracted by medical doctors and certified reviewers and the quality of the French National electronic health data is audited annually by experts. The medical treatments of patients were not available and the deaths occurring in the absence of hospitalization could not be recorded in the database. However, given the age of the population and the total mortality rate (21.3%), it can be assumed that most of the patients presenting a severe and potentially life-threatening disease may have been hospitalized.

## Conclusion

This ten-year observational nationwide study investigated the outcomes of patients who underwent AAA repair in France between 2010–2019. Total in-hospital mortality over the 10-year study was 21.3% and 5.8% of deaths occurred during the first hospital stay for AAA repair. The presence of diabetes was significantly lower in patients with AAA rupture compared to patients with intact AAA. Our results suggested that the presence of diabetes may not worsen immediate post-operative mortality but it may impact survival on longer follow-up period. In multivariate analysis, type 1 diabetes was identified as a risk factor of post-operative mortality after discharge. This study highlights the complex association between diabetes and AAA and should encourage institutions to report long-term follow-up after AAA repair to better understand its impact.

## Supplementary Information

Supplementary Information.
