# Supplementary material for: Nationwide study in France investigating the impact of diabetes on mortality in patients undergoing abdominal aortic aneurysm repair
Source: Sci Rep. 2021 Sep 29;11:19395. doi: 10.1038/s41598-021-98893-x (PMC8481485; doi:10.1038/s41598-021-98893-x)
Supplement: Supplementary file 1 — Supplementary Information. [file 41598_2021_98893_MOESM1_ESM.docx]

**Nationwide study in France investigating the impact of diabetes on mortality in patients undergoing abdominal aortic aneurysm repair**

Juliette Raffort ^1*^, Fabien Lareyre ^2^, Roxane Fabre ^3^, Ziad Mallat ^4^, Christian Pradier ^5^, Laurent Bailly ^5^

^1^ Université Côte d’Azur, CHU, CNRS, Inserm, IRCAN, France

^2^ Department of Vascular Surgery, Hospital of Antibes Juan-les-Pins, France

^3^ CoBTeK lab, Université Côte d’Azur, Nice, France

^4^ Public Health Department, University Hospital of Nice, Université Côte d’Azur, Nice, France.

^5^ Division of Cardiovascular Medicine, University of Cambridge, Cambridge, UK.

**Supplement**

AAA repair was classified into open repair or EVAR according to the Common Classification of Medical Acts (CCAM). The codes identifying patients who had an open repair for a non-ruptured AAA were DGPA001, DGPA005, DGPA008, DGPA010, DGPA012, DGPA013, DGPA016, DGPA017, DGFA015 et DGKA004. Ruptured AAA corresponded to the code DGPA018. Patients who had EVAR were identified using the codes DGLF001, DGLF002 and DGLF005. The additional code I71.3 from the International Classification of Diseases (ICD-10) was used to identify ruptured AAA in this sub-population.

Cardiovascular diseases and metabolic comorbidities were defined according to the International Classification of Diseases (ICD-10) and included arterial hypertension (I10), dyslipidemia (E78.0 to E78.5), type 2 diabetes (T2D) (E11, E12, E13 and E14), type 1 diabetes (T1D) (E10), obesity (E65 and E66), Sleeping Apnea Obstructive Syndrome (SAOS) (G47.3). Congestive heart failure (CHF) corresponded to the codes I50, I11, I13, I09, I25, I42 and I43. A past history of stroke was identified with the codes I60, I61, I62 and I63. Other general comorbidities were collected and included chronic respiratory diseases (J40 to J47) and chronic kidney disease (N18).
